# Small bowel adenocarcinoma of the jejunum detected by double contrast enhanced ultrasound: a case report of a novel ultrasound modality

**DOI:** 10.3389/fonc.2024.1288041

**Published:** 2024-06-19

**Authors:** Shuang Wu, Huizhen Yu, Ying Liu, Hong Zhou, Yang Zhou

**Affiliations:** Department of Medical Ultrasound, Affiliated Hospital of Southwest Jiaotong University, The Third People’s Hospital of Chengdu, Chengdu, Sichuan, China

**Keywords:** small bowel, jejunum, adenocarcinoma, ultrasound, double contrast

## Abstract

**Background:**

Small Bowel Adenocarcinoma (SBA) is rare, occult and life-threatening malignancy in digestive system. Given low incidence and nonspecific symptoms, SBA is frequently detected in later stages. Double contrast enhanced ultrasound (DCEUS) is an innovative imaging technique applied to visualize the gastrointestinal tract, merging intravenous contrast-enhanced ultrasound with oral contrast-enhanced ultrasound. In this case, DCEUS was utilized and successfully detected an SBA of the jejunum.

**Case presentation:**

A Chinese woman, aged 64, sought consultation in the gastroenterology department at our hospital, reporting symptoms of abdominal pain. Three months before entering the hospital, she underwent gastroscopy and colonoscopy which suggested chronic gastritis, and she was treated with oral drugs. However, her symptoms were not relieved, and even worsened. To further investigate, DCEUS was performed. The oral contrast agent dilated the luminal space of the upper gastrointestinal tract, resolving the hindrance caused by gas in the gastrointestinal tract and creating an acoustic window for scanning. Through this acoustic window, oral agent contrast-enhanced ultrasound (OA-CEUS) revealed a localized thickening of jejunal intestinal wall measuring 4x3 cm. Following intravenous injection of ultrasound contrast agent, the jejunal lesion exhibited faster enhancement and heterogeneous hyper-enhancement. Finally, the patient underwent jejunal tumor resection. Pathological examination revealed a jejunal adenocarcinoma.

**Conclusion:**

The timely diagnosis of SBA can be challenging. DCEUS may have the potential to contribute to diagnosis and detailed evaluation of SBA, particularly in cases involving jejunum. Further researches are needed to fully explore the benefits of DCEUS in the standard diagnostic approach for small bowel diseases.

## Introduction

Small bowel adenocarcinoma (SBA) is a rare malignancy of the digestive system. As per the American Cancer Society, small bowel cancers are infrequent when compared to cancers affecting other organs within the digestive system, constituting approximately 3% of digestive system cancers in the year 2022 (1). Small bowel adenocarcinomas make up approximately 30% to 40% of diagnoses of cancer in the small intestine (2). Because of its rarity and concealed nature, SBA is frequently detected in later stages, showing the challenge in identifying this disease and the absence of screening techniques (3). Therefore, detecting the disease as early as possible is important for treatment and prognosis.

Jejunum adenocarcinoma accounts for approximately 18–29% of SBA (4–6), making it an extremely rare type of digestive system cancer. Currently, the only curative treatment for SBA, including jejunum adenocarcinoma, is surgery. However, adenocarcinoma in the jejunum commonly manifests with unusual symptoms, like crampy abdominal pain (4, 7), making it difficult to distinguish from other gastrointestinal disorders. Double contrast enhanced ultrasound (DCEUS) is an innovative technique employed for imaging the gastrointestinal tract, which combines intravenous contrast enhanced ultrasound with oral contrast enhanced ultrasound. The patients orally ingest a contrast agent made from foods like barley, corn, soybeans and yams, which fills and dilates the stomach. This procedure eliminates the hindrance caused by gas in the gastrointestinal tract and establishes a conducive sonographic window for scanning the small bowel, which allows ultrasound to visualize anatomy more clearly and detect lesions easier. With injection of 2.5 ml ultrasound contrast agent made from sulphur hexafluoride microbubbles, lesions can be assessed dynamically from the early arterial phase (10–15s) to the late phase (5 min), with no significant effects on thyroid function (8). Previous research has demonstrated that DCEUS can be a practical substitute for preoperative Borrmann classification of gastric cancer (9). In this particular case, DCEUS was utilized and successfully detected an SBA in the jejunum.

## Case presentation

A woman, aged 64, sought care in the gastroenterology department of our hospital, reporting abdominal pain without apparent predisposing causes. She had a history of cervical cancer and underwent hysterectomy eight years ago. Four years ago, she experienced abdominal pain accompanied by acid reflux and vomiting. Three months prior to admission, she underwent gastroscopy and colonoscopy at local hospital, which suggested chronic gastritis, and she was treated with oral drugs. However, her symptoms of abdominal pain were not completely relieved, and even worsened. Subsequently, she was admitted to our hospital. During this period, her dietary habits and bowel movements were normal, and she did not experience any cessation of gas or stool passage.

The patient has a height of 155cm and a weight of 44kg. On admission, there was a weight loss of 6kg. Her physical examination revealed a heart rate of 55/min, an arterial blood pressure of 132/80 mmHg, a temperature of 36.6°C, and a respiratory rate of 16/min. Blood analysis showed moderate anemia (hemoglobin of 8.5 g/dL) and no leukocytosis.

**Figure 1:**
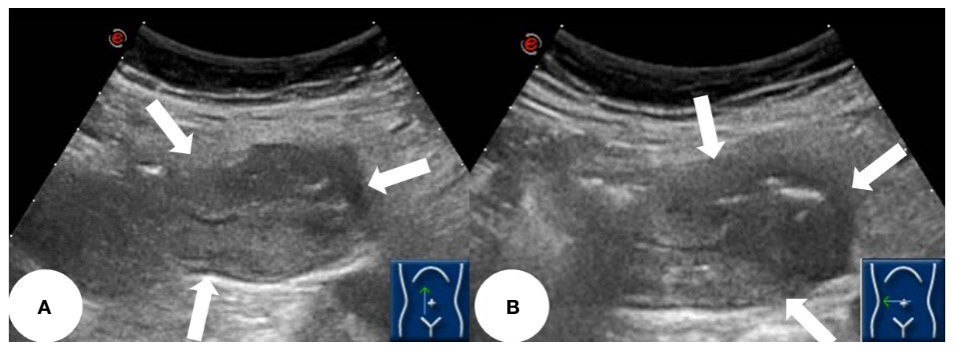
OA-CEUS showing a localized thickening of the jejunal intestinal wall measuring approximately 4x3 cm, with irregular morphology, no longer discernible stratification and involving peri-intestinal fat (white arrows). **(A)** longitudinal section of the lesion. **(B)** transverse section of the lesion.

**Figure 2:**
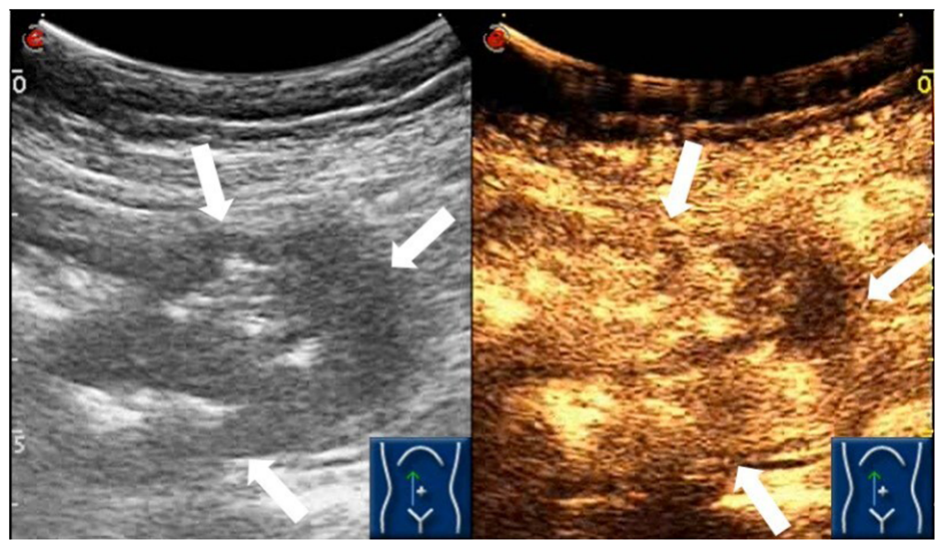
DCEUS demonstrating the abnormal heterogeneous hyper-enhancement of the jejunal intestinal wall (white arrows).

**Figure 3:**
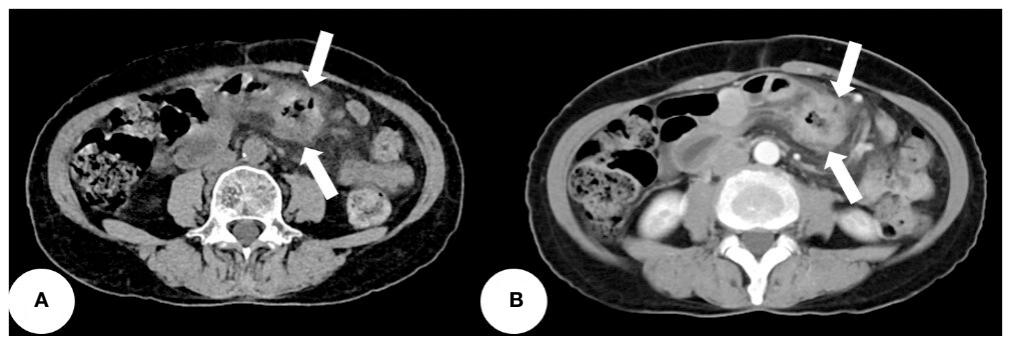
The abdominal CT revealing a thickened jejunal wall resembling a mass, displaying heterogeneous density and enhancement (white arrows). **(A)** Non-contrast CT scan. **(B)** Enhanced CT scan.

Based on the patient’s symptoms, the physician initially suspected gastroesophageal reflux disease (GERD). To further investigate, the patient underwent oral agent contrast-enhanced ultrasound (OA-CEUS) using an Esaote Mylab Twice ultrasound machine (Italy). She ingested 500ml of oral intraluminal contrast agent (Junyi, Yanbian, China). After 40 minutes, the abdominal area was scanned with an ultrasound probe. OA-CEUS revealed reflux of the contrast agent from the stomach to the esophagus. Additionally, the luminal space in the upper gastrointestinal tract was dilated by the oral contrast agent, eliminating the impediment of gas in the digestive tract and providing a sonographic window for scanning. Through this acoustic window, OA-CEUS revealed localized thickening of the jejunal wall, reaching a maximum thickness of approximately 1.5 cm and extending over a length of about 4 cm. The lesion measured 4x3 cm (on ultrasound images, the intestine is composed of an anterior wall and a posterior wall). The original five layers of the intestinal wall completely disappeared, replaced by disorganized hypoechoic patterns, and the lesion’s edges invaded the perienteric fat (**Figure 1**). Following the intravenous injection of 2.5 ml of ultrasound contrast agent (SonoVue, Bracco, Milan, Italy), Double contrast-enhanced ultrasound (DCEUS) was performed. DCEUS allowed real-time distribution of the intravenous contrast agent down to the microcapillary level, aiding in the evaluation of the blood flow within the lesion. The affected intestinal wall enhanced more rapidly than the surrounding normal intestinal wall (at 12s), and the internal region displayed uneven high enhancement, suggesting a potentially rich blood supply to the lesion. The non-enhanced areas might indicate necrotic portions of the tumor (**Figure 2**, **Supplementary Material**). Based on the DCEUS findings, the jejunal lesion was suspected to be malignant. Subsequently, the patient underwent computed tomography (CT) examination. The abdominal CT revealed a thickened jejunal wall resembling a mass, displaying heterogeneous density and enhancement (**Figure 3**).

**Figure 4:**
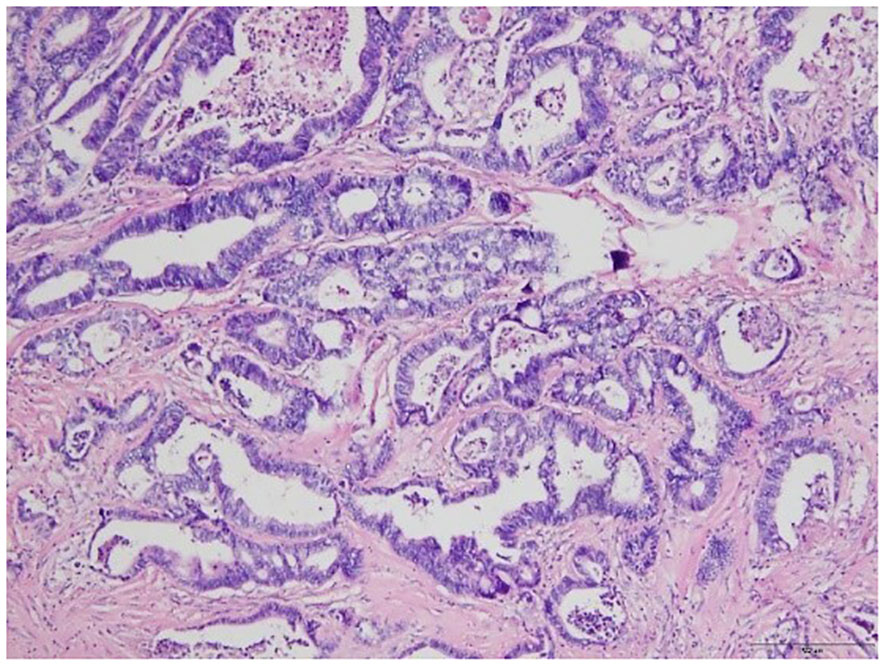
Microscopic image of the tumor from the pathologic specimen; hematoxylin and eosin staining.

**Figure 5:**
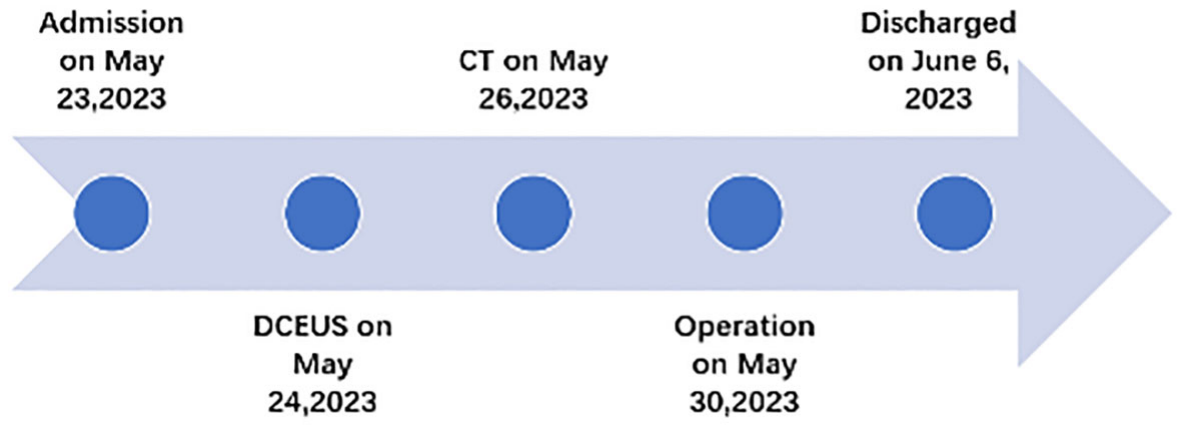
Timeline information in this case report.

Finally, the patient underwent jejunal tumor resection. Intraoperative frozen section pathology examination uncovered an adenocarcinoma in the jejunum. The postoperative gross pathology (**Figure 4**) revealed moderately differentiated adenocarcinoma with no evidence of metastasis in the excised lymph nodes, and successful attainment of a clear surgical margin. The tumor was staged as T4N0M0. After 6 months of follow-up, ultrasound and laboratory tests showed no evidence of local recurrence and metastasis. The timeline of this case is shown in **Figure 5**.

## Discussion

SBA is a rare cancer of digestive system, but its incidence is on the rise (2). The initial symptoms of SBA, such as nonspecific abdominal discomfort, pose challenges for accurate diagnosis, as they can overlap with symptoms of other digestive diseases. Consequently, SBA is often diagnosed at more advanced stages compared to colorectal cancer (3). Additionally, SBA frequently presents with complications in the immediate area, including obstruction (40%) or bleeding (26%), further complicating its management (4, 10). One study indicated that outcomes of SBAs appeared not to have improved with the progress of technology and time (10). The explanation for this finding may be the lack of screening methods, resulting in missed opportunities for timely operative treatment.

Predisposing diseases for SBA include Familial Adenomatous Polyposis (FAP), Hereditary Non-Polyposis Colorectal Cancer (HNPCC) syndrome, Peutz-Jeghers syndrome, Crohn’s disease and celiac disease (11). In individuals with these underlying factors, consideration should be given to examining the small bowel through enteroscopy, enteroscan or videocapsule (11). While gastroscopy and enteroscopy are suitable for tumors situated in proximity to the proximal duodenum or terminal ileum (12). Video capsule endoscopy (CE) or double-balloon enteroscopy (DBE) are more effective for evaluating the rest of the small bowel. Several research has documented the secure and efficient utilization of DBE, especially for patients with small bowel strictures (13–16). On the other hand, CE provides a more comprehensive inspection of the complete small bowel mucosa; however, it is not favored because of the challenges in obtaining biopsy tissue for diagnosis and complications in cases of obstruction or stricture (17–19). For intestinal diseases, CE has been reported to yield a definitive diagnosis in only 20–30% of cases, whereas DBE accounts for 60–70% (20). CE is not easily repeatable, and its images are transient and random, due to uncontrollable movement direction and speed. Furthermore, intestinal cleanliness and gastrointestinal motility significantly impact the quality of the images. It is challenging for CE to differentiate submucosal lesions without surface erosions or ulcers from the pressure exerted by external lesions (21, 22). Additionally, CE tends to misdiagnose larger tumors with erosive lesions as local inflammation, resulting in missed tumor detection (23, 24). Compared to CE, DBE is more uncomfortable and has a lower completion rate, leading to a reduced probability of detecting small bowel diseases. Moreover, the presence of intestinal mucosal folds can make it difficult to observe certain lesions for DBE (20).

According to the NCCN guideline, cross-sectional imaging modalities are also essential during the initial examination of SBA to assess local tumor invasion, such as CT and magnetic resonance imaging (MRI) (3). However, conventional radiological imaging has limited sensitivity, which can lead to delayed diagnosis for patients (25, 26). Furthermore, CT also has some limitations, including exposure to ionizing radiation, which is particularly concerning for the pediatric population, as well as the need for intravenous iodinated contrast material, carrying intrinsic dangers of unfavorable reactions and prospective complications (26). MRI appears to offer greater accuracy than CT in the diagnosis of small bowel diseases. A prospective investigation involving 150 patients suspected of small bowel disease found that MRI demonstrated superior accuracy compared to CT, especially for neoplastic diseases (P=.0412) (27). Despite its diagnostic advantages, MRI has some drawbacks, including significant noise, extended duration of the examination, confined space for the patient, relatively low availability in certain regions, higher cost, and motion artifacts due to intestinal peristalsis.

Ultrasound, especially CEUS has been gaining popularity for small bowel disease in the last two decades (28). This case report details an uncommon occurrence of jejunum adenocarcinoma in a 64-year-old Chinese woman identified through DCEUS. To the best of our knowledge, this represents the initial documentation of SBA identified through ultrasound. Ultrasound can visualize the five layers of the normal intestinal wall, including, from inner to outer layers, the hyperechoic mucosal epithelium, hypoechoic mucosal muscle layer, hyperechoic submucosal layer, hypoechoic muscularis propria, and hyperechoic serosal layer. In this case, we identified localized thickening of the jejunal wall, reaching a maximum thickness of approximately 1.5 cm and extending over a length of about 4 cm. The lesion measured 4x3 cm(on ultrasound images, the intestine is composed of an anterior wall and a posterior wall). The original five layers of the intestinal wall completely disappeared, replaced by disorganized hypoechoic patterns, and the lesion’s edges invaded the perienteric fat. Following the administration of intravenous contrast, the affected intestinal wall enhanced more rapidly than the surrounding normal intestinal wall, and the internal region displayed uneven high enhancement, suggesting a potentially rich blood supply to the lesion. The non-enhanced areas might indicate necrotic portions of the tumor. Based on these findings, we suspect adenocarcinoma of the small intestine. Moreover, in this case, DCEUS revealed tumor penetration beyond the serosal layer, involving the perienteric fat, aligning with pathological staging as T4.

DCEUS offers distinct advantages compared to conventional sectional imaging modalities. One is its repeatability, as it can be performed repeatedly at the bedside without subjecting the patient to ionizing radiation. By utilizing oral and intravenous contrast agents, DCEUS enables real-time dynamic observation of the gastrointestinal organs from different angles. This real-time imaging capability, when performed by an experienced sonographer, can result in excellent diagnostic value for small bowel diseases. DCEUS is convenient, cost-effective, radiation-free, and offers good repeatability, making it more readily accepted by patients. After the patient ingests oral contrast agent, which is made from foods like barley, corn, soybeans, and yams and poses no health risks, ultrasound effectively displays the gastrointestinal tract by reducing interference from digestive gas. This makes ultrasound a valuable screening tool for gastrointestinal diseases. Additionally, ultrasound intravenous contrast agents have no renal toxicity, making them suitable for patients with compromised kidney function. In contrast, enhanced CT is relatively more expensive, involves ionizing radiation, and carries the risk of contrast-induced nephrotoxicity, imposing certain limitations. Furthermore, ultrasound provides clear visualization of the five layers of the intestinal wall, allowing for accurate assessment of tumor local infiltration. It can perform T-staging with reasonable accuracy; for instance, in this case, ultrasound revealed tumor penetration beyond the serosal layer, involving the perienteric fat, aligning with pathological staging as T4.

In this reported case, DCEUS played a crucial role in the diagnosis of SBA involving the jejunum. The DCEUS findings revealed an abnormally thickened jejunal wall and its enhanced characteristics, leading to the suspicion of SBA.

## Conclusion

SBA of jejunum is very rare, occult and life-threatening malignancy in digestive system. Given its low incidence and nonspecific symptoms, timely diagnosis can be challenging. DCEUS offers several advantages, including being noninvasive, convenient, inexpensive, and radiation-free. It may have the potential to significantly contribute to diagnosis and detailed evaluation of SBA, particularly in cases involving the jejunum. Further research and clinical studies are required to thoroughly investigate the benefits of DCEUS and to establish its place in the standard diagnostic approach for small bowel diseases, ultimately improving patient outcomes and prognosis.

## Data availability statement

The original contributions presented in the study are included in the article/**Supplementary Material**. Further inquiries can be directed to the corresponding author.

## Ethics statement

Written informed consent was obtained from the individual(s) for the publication of any potentially identifiable images or data included in this article.

## Author contributions

SW: Writing – original draft, Writing – review & editing. HY: Writing – review & editing. YL: Writing – review & editing. HZ: Writing – review & editing. YZ: Formal analysis, Supervision, Writing – review & editing.
